# Simulating the Overall Hospital Quality Star Ratings With Random Measure Weights

**DOI:** 10.1001/jamanetworkopen.2025.19029

**Published:** 2025-07-03

**Authors:** Benjamin D. Pollock, Daniel S. Ubl, Subashnie Devkaran, Sean C. Dowdy

**Affiliations:** 1 Robert D. and Patricia E. Kern Center for the Science of Health Care Delivery, Division of Health Care Delivery Research, Mayo Clinic, Jacksonville, Florida; 2 Robert D. and Patricia E. Kern Center for the Science of Health Care Delivery, Mayo Clinic, Rochester, Minnesota; 3 Department of Quality and Value, Mayo Clinic, Rochester, Minnesota

## Abstract

**Importance:**

Hospital ratings including the *US News & World Report*’s Best Hospitals rankings and the Centers for Medicare & Medicaid Services’ (CMS’) Overall Hospital Quality Star Rating (Overall Star Rating) measure different outcomes and are weakly correlated. Therefore, methods for defining and measuring reliable excellence, defined as consistently great performance across all quality measures, are needed.

**Objective:**

To assess a measure of reliable excellence using the 45 quality measures reported in the Overall Star Ratings.

**Design, Setting, and Participants:**

This cross-sectional study used hospital-level data from the 2023 and 2024 CMS Overall Star Ratings at all US hospitals with a 2023 and 2024 CMS Overall Star Rating.

**Exposures:**

The exposure was the CMS Overall Star Rating summary score, a continuous variable calculated from the weighted *z* scores of 45 quality measures used in the Overall Star Rating. A total of 100 000 simulations were run in which all US hospitals’ CMS Overall Star Rating summary scores were calculated through summation of *z* scores from randomly generated measure weights, as opposed to the existing weights used in the Overall Star Rating method.

**Main Outcomes and Measures:**

Reliable excellence, defined as achieving a 90th percentile (or better) CMS Overall Star Rating summary score on at least 50 000 of 100 000 simulations. The percentage of hospitals achieving reliable excellence was calculated both overall and stratified by CMS Ovearll Star Ratings.

**Results:**

There were 2700 hospitals in the analysis, with 335 5-star hospitals (12.4%), 727 4-star hospitals (26.9%), 799 3-star hospitals (29.6%), 572 2-star hospitals (21.2%), and 267 1-star hospitals (9.9%) in the 2024 CMS Overall Star Rating. A total of 244 of 2700 hospitals (9.0%) met the study definition of reliable excellence, whereas 1287 of 2700 hospitals (47.7%) achieved excellence in at least 1 simulation.

**Conclusions and Relevance:**

This cross-sectional study of 2700 US hospitals found that only 244 hospitals (9.0%), including less than two-thirds of the CMS 5-star rated hospitals, were reliably excellent across 100 000 CMS Overall Star Rating scoring simulations using random measure weightings. These findings lend credence to the ubiquity of inconsistent greatness in health care quality and illuminate the need for methods to distinguish hospitals that provide reliably excellent care.

## Key Points

**Question:**

Can a measure of reliable excellence in hospital quality be developed from simulating the Centers for Medicare & Medicaid Services Overall Hospital Quality Star Rating summary score using random measure weights?

**Findings:**

In this cross-sectional study of 2700 US hospitals, 9.0% met the definition of reliable excellence, including 61.8% of 335 5-star rated hospitals, whereas 47.7% of all hospitals achieved excellence in at least 1 simulation.

**Meaning:**

These findings suggest meaningful variation in hospital quality performance even among 5-star hospitals, and the measure of reliable excellence complements the Overall Hospital Quality Star Ratings by distinguishing hospitals likely to provide top-decile quality regardless of measure weights.

## Teaser

This cross-sectional study assesses an approach to define and distinguish reliable excellence across all individual quality measures of the Centers for Medicare & Medicaid Services Overall Hospital Quality Star Rating.

## Introduction

In a 2023 interview in *NEJM Catalyst Innovations in Care Delivery*, Dr John Phipps and Editor-in-Chief Dr Thomas Lee discussed the concept of “inconsistent greatness” in health care delivery.^1^ Inconsistent greatness encompasses the notion that US health care delivery systems are challenged with ever-evolving quality performance benchmarks across multiple domains of hospital quality, including patient safety, patient experience, value, and system or process measures. Varying domains of quality measurement lead to inconsistent greatness, with many hospitals achieving excellent performance or meeting benchmarks for some quality measures while experiencing poor performance for other measures. The evidence supports this theme of inconsistent greatness, recognizing that high-profile hospital quality rankings and ratings, including the *US News & World Report*’s (*USNWR*) Best Hospitals rankings, the Centers for Medicare & Medicaid Services’ (CMS’) Overall Hospital Quality Star Rating (Overall Star Rating), and the Leapfrog Hospital Safety Grade, measure different outcomes and are weakly correlated.^2,3^ This is not surprising because these rankings and ratings have been intentionally developed with foci on different domains of quality. The Leapfrog Hospital Safety Grade focuses on patient safety and the *USNWR* Best Hospitals focuses on 30-day mortality and reputation among physicians, whereas the CMS Overall Star Rating is a more general rating inclusive of multiple quality domains.^4^ However, these differences in methods and purposes lead to discrepancies that can be difficult to interpret. For example, many hospitals that are top-ranked regionally by *USNWR* fail to achieve 5-star CMS ratings.^5^ This discordance makes hospital quality reporting confusing for both patients and hospital leaders and neatly illustrates the concept of “inconsistent greatness” in health care delivery.^6^

To overcome “inconsistent greatness,” we must develop methods for defining, detecting, and measuring its counterpart: reliable excellence. Reliable excellence can be conceptualized as consistently great performance over time across the full spectrum of quality measures, methods, and patient outcomes. As a starting point, we recognize that some hospitals deliver more reliably excellent care and quality than others. However, we currently lack methods and reporting tools to define, identify, and learn from reliably excellent performers. As a result, lessons regarding processes, cultures, or systems leading to reliable excellence get lost in the noise of inconsistent greatness and may go unshared to the detriment of patients and payers. This was particularly true during the years of the COVID-19 pandemic, at which time quality and safety scores were suppressed rather than risk adjusted, missing an opportunity to learn from the most robust health care systems.^7,8^

A previous report^4^ described an approach to composite hospital quality measurement, in which percentile scores from distinctly different hospital quality rankings and ratings were aggregated to set benchmarks for consistently high performance across multiple methods within a health system. However, even when an aggregate approach was used, top-performing hospitals may be inconsistently great across individual measures that are not weighted heavily enough to impact the overall rating. Because these statistical weighting schemes can be subjective, they contribute to inconsistent greatness; hospitals can perform differently when scores are reweighted using the identical set of measures.^9^

To define and evolve the detection of reliable excellence, we must explore variability in hospital performance across individual quality measures without being limited by the weighting method. Some hospital rating measure weights, including those used in the CMS Overall Star Ratings, are based on clinical and methodology expert consensus. However, this approach can still result in measure weights that differ from year to year or contain some amount of subjectivity. Nevertheless, the robustness and ubiquity of the CMS Overall Star Ratings make them a useful framework within which to test a new, potentially relevant indicator of consistency across all quality measures and domains, regardless of the weighting method used, which we call reliable excellence. In this study, we assessed an approach to define and distinguishing reliable excellence across all individual quality measures of the CMS Overall Star Rating spanning the domains of readmissions, mortality, patient safety, patient experience, and timeliness and effectiveness of care.

## Methods

### Study Population

We began with the population of 2811 US hospitals that received both a 2024 and 2023 CMS Overall Star Rating.^10^ We then excluded 111 (3.9%) acute care Veterans Affairs (VA) hospitals (3.9%). Although VA hospitals became eligible to receive Star Ratings for the first time in 2023, incongruities in data availability and measure completeness remain for VA hospitals. As such, our final study sample included 2700 US hospitals. The Mayo Clinic institutional review board (IRB) confirmed that this study was not human subjects research and therefore did not need IRB review or informed consent, in accordance with 45 CFR 46.102. This study followed the Strengthening the Reporting of Observational Studies in Epidemiology (STROBE) reporting guidelines for cross-sectional studies.^13^

### Study Design and Analyses

In this analysis, we defined excellence as performance at or above the 90th percentile nationally, which is consistent with our previously described performance thresholds for hospital rankings and ratings.^4^ This distribution (ie, top 10%) is roughly the same threshold for achieving 5 Stars in the CMS Overall Star Rating system, with 8% of US hospitals achieving 5-star Ratings in 2024.^11^ We assessed the hospital-level distribution of the total count (of 45) of CMS Overall Star Ratings in which hospitals achieved 90th percentile performance. Then using the 45 individual measure *z* scores from the 2023 and 2024 CMS Overall Star Rating SAS Packs,^12^ we ran 100 000 simulations separately per year (2023 and 2024) in which each year’s CMS Overall Star Rating summary score was calculated through the summation of all measure *z* scores with randomly generated weights, with all weights being greater than 0 and less than 1, such that the sum of the 45 measure weights equaled 1:

CMS Overall Star Rating Summary Score = Sum [(Measure_1_ × Random Weight_1_) + (Measure_2_ × Random Weight_2_) … + (Measure_45_ ×Random Weight_45_)]

Within each of 100 000 simulations, a hospital was labeled as excellent if its Overall Star Rating summary score was among the top 10 percentiles (ie, ≥90th percentile) of summary scores for that simulation. We then calculated the percentage of simulations (of 100 000) in which each hospital achieved excellence. Any hospital that achieved excellence in at least 50 000 of 100 000 simulations (50.0%) was determined to have achieved reliable excellence. The percentage of hospitals achieving reliable excellence was calculated both overall and stratified by CMS Overall Star Ratings. We conducted a sensitivity analysis to assess the number of hospitals that would achieve reliable excellence at an 80th percentile performance threshold (instead of 90th percentile). We also conducted a sensitivity analysis to determine the number of hospitals that would achieve reliable excellence (at the original 90th percentile performance threshold) using different simulation percentage thresholds (eg, 10% of simulations instead of 50% of simulations). We ran additional sensitivity analyses to assess variation in the total number of hospitals deemed reliably excellent across 1000, 5000, 50 000, 100 000, 250 000, and 500 000 simulations as well as the intrahospital variation in performance within each amount of simulations to assess the robustness in capturing an effective sample. We also performed a κ analysis comparing the 2023 and 2024 data to assess the variation in hospitals’ reliable excellence statuses compared with the variation in hospitals’ CMS 5-star Ratings from 2023 to 2024.

### Statistical Analysis

All analysis was conducted using SAS software, version 9.4 (SAS Institute Inc). A 2-sided *P* < .05 was considered statistically significant.

## Results

A total of 2700 hospitals were included in the analysis, with 335 5-star hospitals (12.4%), 727 4-star hospitals (26.9%), 799 3-star hospitals (29.6%), 572 2-star hospitals (21.2%), and 267 1-star hospitals (9.9%) in 2024 (Table). Among all hospitals in 2024, none achieved 90th percentile performance on 50.0% or more of the CMS Overall Star Rating measures (eTable 3 and eFigure 1 in Supplement 1). The maximum number of CMS Overall Star Rating measures in which any hospital was at the 90th percentile or above was 20 of 45 measures, and the median (IQR) was 3 (2-5). Among 100 000 simulations with randomly generated measure weightings, 207 of 335 5-star hospitals (61.8%) performed at or above the 90th percentile in at least 50 000 of 100 000 simulations (50.0%) to achieve the study definition of reliable excellence, along with 34 of 727 4-star hospitals (4.7%) and 3 of 799 3-star hospitals (0.4%) (Table). Combined, this resulted in 244 of 2700 hospitals (9.0%) meeting the definition of reliable excellence. Among 2700 hospitals, 1287 (47.7%) achieved excellence in at least 1 simulation (eTable 1 in Supplement 2). Hospital-specific summary results, including the mean, median, range, and 5th, 25th, 75th, and 95th percentile summary scores across 100 000 simulations for all 2700 included hospitals are available in eTable 1 in Supplement 2. The descriptive statistics and individual random measure weights generated in each of the 100 000 simulations are given in eTable 2 in Supplement 2.

**Table.** Simulated Centers for Medicare & Medicaid Services Overall Hospital Quality Star Rating Summary Score Percentiles Aggregated by Actual 2024 Star Rating^a^

Result across 100 000 simulations	5-Star hospitals (n = 335)	4-Star hospitals (n = 727)	3-Star hospitals (n = 799)	2-Star hospitals (n = 572)	1-Star hospitals (n = 267)
Percentile, mean (SD)	89.4 (7.3)	69.8 (12.0)	45.4 (14.1)	24.8 (15.2)	9.5 (11.1)
Percentile, median (IQR)	90.6 (85.5-94.9)	70.1 (61.8-77.9)	43.8 (34.8-53.6)	20.8 (14.3-30.2)	6.5 (3.3-10.1)
Percentile, range	53.5-99.0	28.8-97.5	11.4-96.4	4.0-87.8	0.0-72.6
Hospitals meeting the study definition of reliable excellence, No. (%)^b^	207 (61.8)	34 (4.7)	3 (0.4)	0	0

^a^ Using random weights for all measures underlying the summary score calculation (see eTable 2 in Supplement 1 for detailed data regarding the random weights used in each simulation).

^b^ At least 50 000 of 100 000 simulations (50.0%) at the 90th percentile summary score or above.

Of the 244 reliably excellent hospitals, 143 (58.6%) were reliably excellent when this method was applied to the 2023 Overall Star Ratings, with κ indicating moderate agreement in reliable excellence status between years (0.54; 95% CI, 0.48-0.59; *P* < .001). The κ agreement in the CMS 5-star status comparing 2024 vs 2023 was 0.57 (95% CI, 0.53-0.62; *P* < .001).

In the first sensitivity analysis, 507 hospitals (18.8%) achieved reliable excellence when the performance threshold was lowered to the 80th percentile (instead of the 90th percentile). The number of hospitals that would achieve reliable excellence at the original 90th percentile performance threshold using different simulation percentage thresholds is shown in eFigure 2 in Supplement 1. In the additional sensitivity analyses, the number of reliably excellent hospitals remained stable, ranging from 237 to 245 across all numbers of simulations from 1000 to 500 000 simulations. Notably, the maximum intrahospital difference among reliably excellent hospitals comparing the 25th and 75th percentiles across 100 000 simulations was 12 percentile points (84% vs 96%) (eTable 1 in Supplement 2).

## Discussion

In this analysis, substantially fewer US hospitals met the definition of reliable excellence than achieved CMS 5-Star Ratings. Only 244 US hospitals achieved reliable excellence when defined as 90th percentile performance or better in at least 50.0% of simulated CMS Overall Star Rating summary scores using random measure weightings (Figure). In layperson’s terms, 244 hospitals are likely to perform in the top decile in the CMS Overall Star Rating regardless of the weights used for each measure. Interestingly, approximately half of hospitals achieved excellence in at least 1 of 100 000 simulations, suggesting the potential for occasional excellence (90th percentile or better performance) to be found at nearly half of US hospitals as a statistical artifact of measure weight selection rather than a reflection of true excellence or quality. Taken together, these findings lend credence to the current ubiquity of inconsistent greatness^1^ in health care quality and speak to the need for better methods to define and detect reliable excellence.

**Figure.**
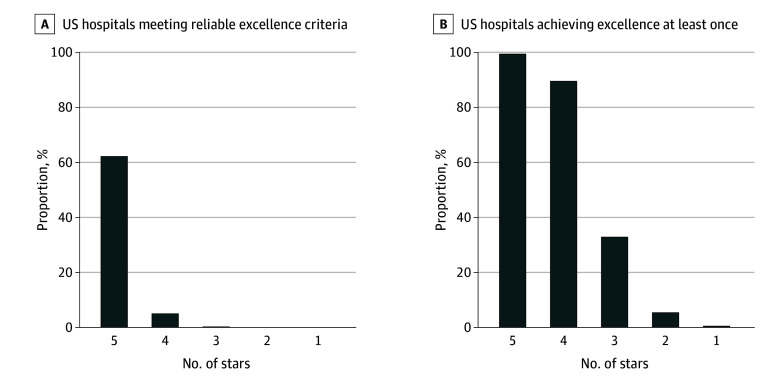
Percentage of US Hospitals That Met the Reliable Excellence Criteria vs Those That Achieved Excellence at Least Once, Stratified by Actual 2024 Star Rating Reliable excellence is defined as a score in the 90th percentile or higher in at least 90% of 100 000 Centers for Medicare & Medicaid Services (CMS) Overall Star Rating simulations using random measure weightings. Achieving excellence at least once is defined as being in the 90th percentile or higher in at least 1 of 100 000 CMS Overall Star Rating simulations using random measure weightings.

For decades, the aim of quality improvement has been to work toward complete avoidance of errors, which is often conceptualized as a zero harm guiding principle.^14^ After decades of patient safety measurement and improvement work to reach this goal, results suggest that a zero harm target, an important aspirational goal, may be impractical and unattainable in the near term. Bates et al^15^ reported that nearly 1 in 4 hospital patients experience harm in the inpatient setting, and data from Levine et al^16^ reported a 7% rate of adverse events in the outpatient setting. Instead, it may be prudent to shift our focus toward promoting a paradigm of reliable excellence in which hospitals seek to meet or exceed benchmarked targets across the entire spectrum of adverse events and outcomes consistently over time. This paradigm would include top decile performance in quality and safety (eg, infections, falls, pressure ulcers, readmissions, and mortality) in addition to patient experience. In other words, instead of a hyperfocus and dedication of disproportionate hospital resources toward prevention of a single catheter-associated urinary tract infection for achieving zero harm, we should instead strive to reliably meet achievable benchmarks of excellence in all measures of importance to patients year after year.

A reliably excellent hospital must perform at a high level across all domains and measures of quality, with a clear vision toward putting the patient first. Building and maintaining a steadfast commitment to proven processes and a culture of safety over time, as opposed to a once-a-year prioritization or quick-fix project to improve a single quality measure, such as catheter-associated urinary tract infection rates, is likely the foundation for reliably excellent health care. To their credit, hospital rankings and ratings, such as the CMS Overall Star Ratings and the *USNWR* Best Hospitals rankings inherently incorporate the time component of reliable excellence because they generally include 2 to 5 years of aggregate patient outcomes data in their calculations.^10,17^ However, in the absence of definitions and methods to distinguish reliable excellence, we lack the precision to determine where in the US health care environment to begin our search for reliably excellent processes and cultures.

Our analysis is not able to provide an explanation or specific insight as to how 244 hospitals achieved reliable excellence but rather serves as a first step to suggest a potential approach for detecting reliable excellence across the spectrum of existing quality measures. Our measurement framework is advantageous because of its avoidance of the CMS Overall Star Rating measure weightings, which, although drawn from evidence-based expert panel discussions, may still harbor some unavoidable level of subjectivity. In fact, difficult decisions regarding domain and measure weightings are currently a feature of all hospital rankings and ratings. Likewise, an advantage of our approach was its reliance on readily available and endorsed quality measures from the CMS Overall Star Rating.

### Limitations

Limitations of the analysis include those intrinsic to our selection of the CMS Overall Star Rating as a framework for exploring reliable excellence. The CMS Overall Star Rating consists of rigorously developed, validated, and endorsed quality measures. Nevertheless, the CMS Overall Star Ratings may lack some domains of quality that are meaningful to patients, such as long-term survival, functional abilities, and health equity. Other criticisms often lobbed at the CMS Overall Star Ratings include those that tend to hamper all hospital rankings and ratings: year-to-year volatility and small event sizes, limiting the applicability and availability of certain quality measures at low-volume hospitals, inconsistent peer grouping methods for interhospital comparisons, and the timeliness and transparency of the data, which often lags by several years and can be difficult for patients to interpret. Additionally, using random weightings may not be an ideal approach for quality indexes, and with billions of possible random weighting combinations, it is possible our approach did not adequately sample the full set of possibilities, even with 500 000 simulations. However, we saw significant convergence and narrow hospital performance ranges across increasing numbers of simulations. Furthermore, the same 244 hospitals that achieved reliable excellence in the main analysis were also reliably excellent at 250 000 and 500 000 simulations, with 1 additional hospital achieving reliable excellence after 250 000 and 500 000 simulations. The distributions and descriptive statistics of random measure weights across 100 000 simulations are further detailed in eTable 2 in Supplement 1.

Another limitation is that our approach arbitrarily caps reliable excellence at 540 hospitals (100 000 simulations × 270 top 10 percentile hospital spots per simulation = 27 000 000 total hospital spots available; 27 000 000 of 50 000 hospital spots needed to achieve reliable excellence = 540 hospitals), whereas the CMS 5-star Rating is capped statistically through *k*-means clustering, which typically results in fewer than 500 5-star hospitals each year. Thus, the intent of the reliable excellence indicator is not to compare directly with 5-star hospitals but rather to uniquely identify hospitals that have comparatively better performance across a wider range of measure weightings. By selecting the 50% simulation threshold in our main analysis, reliable excellence can be practically interpreted as describing any hospital that is more likely than not to perform at or above the 90th percentile in the CMS Overall Star Ratings regardless of the measure weights. Furthermore, our κ analysis showed that reliable excellence status between 2023 and 2024 varied roughly the same amount as a CMS 5-star Rating, indicating our reliable excellence status has comparable robustness from year to year.

We also acknowledge there may be merit in using unequally weighted individual measures in quality indexes when these weights are based empirically or clinically on rigorous expert panel discussion patients’ care preferences or relative harm scales. To this point, our selection of the CMS Overall Star Rating as the foundation on which to explore reliable excellence reflects its inherent relative validity. Nevertheless, our analysis indicated the substantial variability across the existing CMS Overall Star Rating landscape; no hospitals achieved 90th percentile or greater performance in even half of the measures. Moving forward, the measurements of hospital quality and reliable excellence will need to evolve to find ways to incorporate individual patient preferences regarding the quality outcomes of most importance. Future approaches to assess reliable excellence could also expand the scope beyond the measures used in the CMS Overall Star Rating to incorporate a variety of additional measures, which could be facilitated by use of the CMS Care Compare Application Programming Interface to assess more than 150 quality measures in the CMS Inpatient Quality Reporting and Outpatient Quality Reporting programs.^18^

## Conclusions

We found that only 244 US hospitals achieved reliable excellence in hospital quality in 2024 when defined as 90th percentile performance or better in at least 50.0% of 100 000 simulations using random weights for each measure in the CMS Overall Star Ratings. Our analysis highlights that there is meaningful variation in hospital quality performance across the spectrum of quality measures, even among 5-star hospitals. Future efforts to assess this variation may allow for better identification of reliably excellent hospitals, which could in turn lead to solicitation of evidence regarding the processes or cultures that separate reliable excellence from inconsistent greatness in hospital quality.

## Notes

Supplement 1. **eFigure 1. **Hospital-Level Distribution of Total Measures (Out of 45) in 90th Percentile or Above (N=2,700) **eFigure 2. **Total Number of Hospitals Which Would Be Deemed Reliably Excellent (90th Percentile or Above) Based on Simulation Threshold **eTable 3.** Descriptive Statistics for Number of Measures Reliable Excellent Per Hospital (N=2,700)

Supplement 2. **eTable 1. **Hospital-Specific Summary Results **eTable 2. **Descriptive Statistics and Individual Random Measure Weights

Supplement 3.
Data Sharing Statement
