# Supplementary material for: Simulating the Overall Hospital Quality Star Ratings With Random Measure Weights
Source: JAMA Netw Open. 2025 Jul 3;8(7):e2519029. doi: 10.1001/jamanetworkopen.2025.19029 (PMC12232183; doi:10.1001/jamanetworkopen.2025.19029)
Supplement: Supplement 1. — eFigure 1. Hospital-Level Distribution of Total Measures (Out of 45) in 90th Percentile or Above (N=2,700) eFigure 2. Total Number of Hospitals Which Would Be Deemed Reliably Excellent (90th Percentile or Above) Based on Simulation Threshold eTable 3. Descriptive Statistics for Number of Measures Reliable Excellent Per Hospital (N=2,700) [file jamanetwopen-e2519029-s001.pdf]

Supplemental Online Content

Pollock BD, Ubl DS, Devkaran S, Dowdy SC. Simulating the overall hospital quality star ratings with random measure weights. *JAMA Netw Open*. 2025;8(7):e2519029. doi:10.1001/jamanetworkopen.2025.19029

eFigure 1. Hospital-Level Distribution of Total Measures (Out of 45) in 90th Percentile or Above (N=2,700)

eFigure 2. Total Number of Hospitals Which Would Be Deemed Reliably Excellent (90th Percentile or Above) Based on Simulation Threshold

eTable 3. Descriptive Statistics for Number of Measures Reliable Excellent Per Hospital (N=2,700)

This supplemental material has been provided by the authors to give readers additional information about their work.

© 2025 Pollock BD et al. *JAMA Network Open*

eFigure 1. Hospital-Level Distribution of Total Measures (Out of 45) in 90th Percentile or Above (N=2,700)

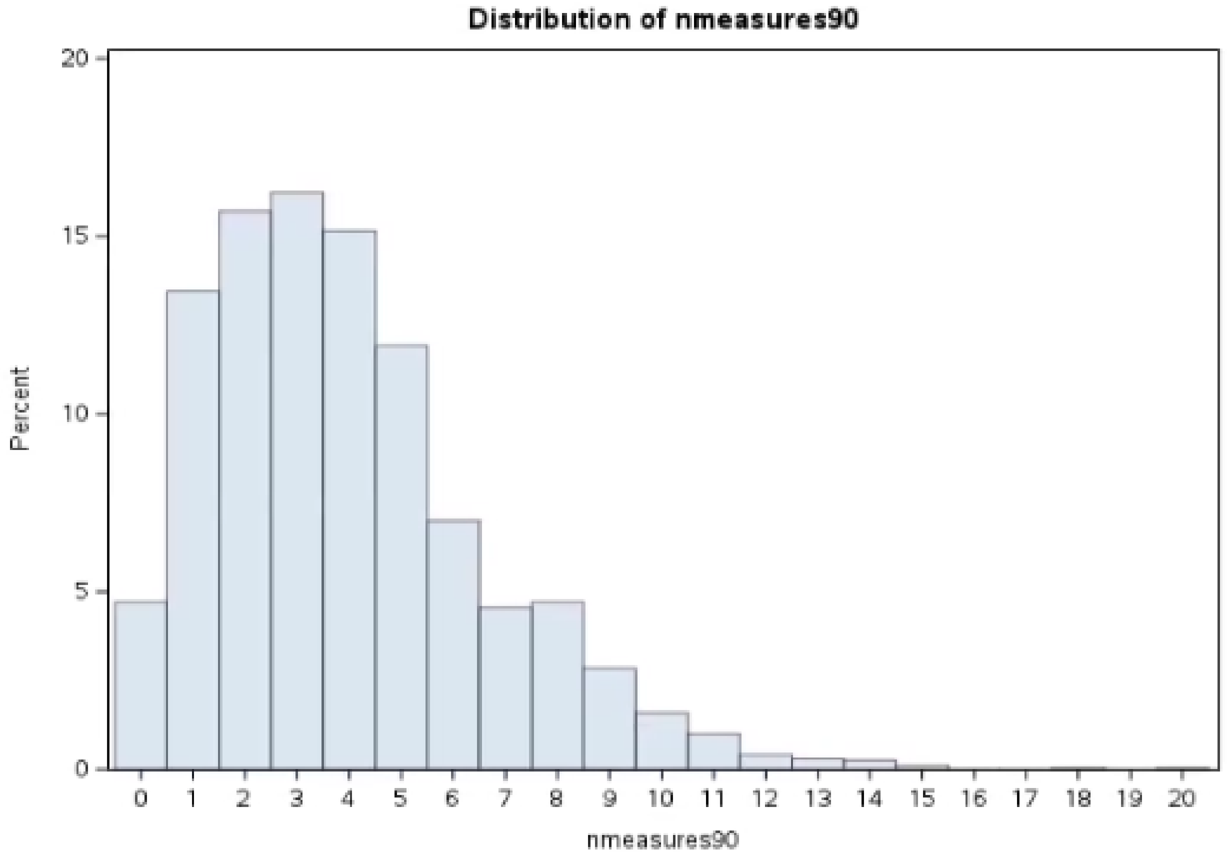

© 2025 Pollock BD et al. *JAMA Network Open*

eFigure 2. Total Number of Hospitals Which Would Be Deemed Reliably Excellent (90th Percentile or Above) Based on Simulation Threshold

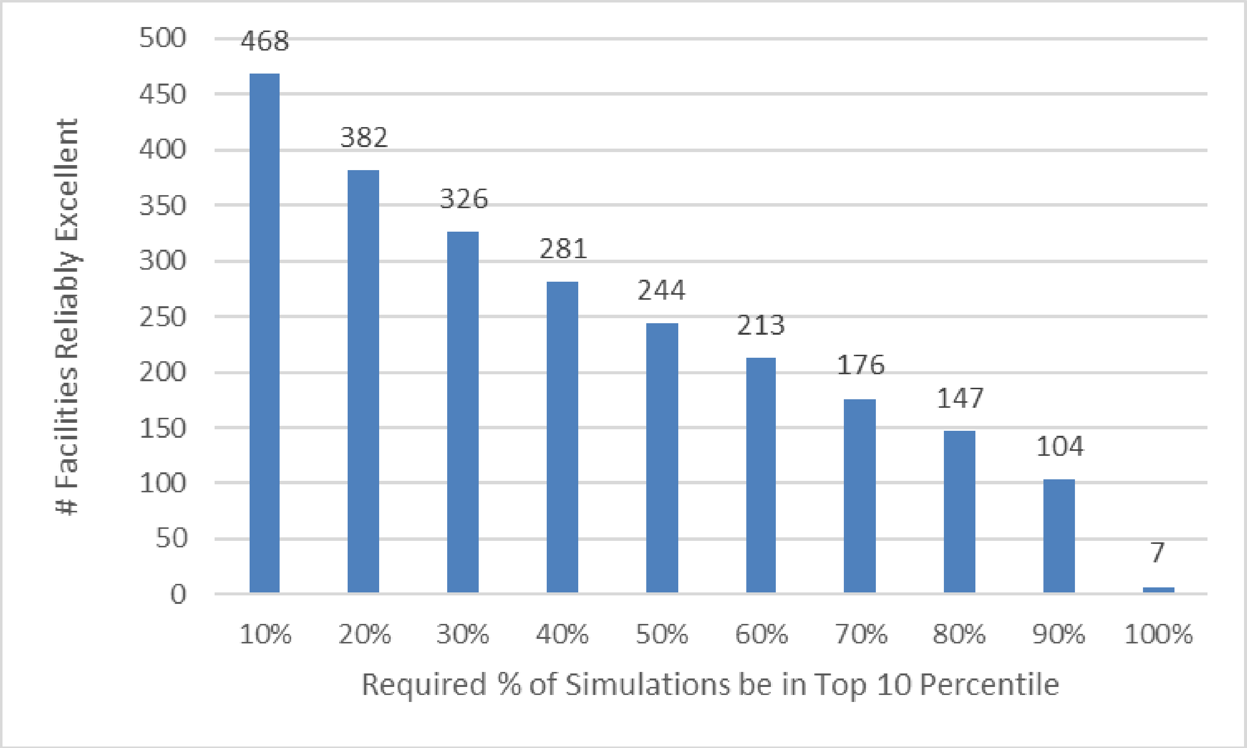

In the main analysis, there were 244 reliably excellent hospitals with the simulation threshold set at 50% of simulations (50,000/100,000 simulations). There were 7 hospitals which were reliably excellent in 100% (100,000/100,000) of simulations.

© 2025 Pollock BD et al. *JAMA Network Open*

eTable 3. Descriptive Statistics for Number of Measures Reliable Excellent Per Hospital (N=2,700)

**Percentile**	**Number of measures (out of 45) reliably excellent (90th percentile or better)**
100 -Max	20
99	12
95	9
90	8
75	5
50	3
25	2
10	1
5	1
1	0
0 - Min	0
**n**	2,700
**Mean**	3.93
**Median**	3
**Mode**	3
**Std Deviation**	2.67

© 2025 Pollock BD et al. *JAMA Network Open*
