# Supplementary material for: Simulating the Overall Hospital Quality Star Ratings With Random Measure Weights
Source: JAMA Netw Open. 2025 Jul 3;8(7):e2519029. doi: 10.1001/jamanetworkopen.2025.19029 (PMC12232183; doi:10.1001/jamanetworkopen.2025.19029)
Supplement: Supplement 3. — Data Sharing Statement [file jamanetwopen-e2519029-s003.pdf]

Data Sharing Statement

Pollock. Simulating the Overall Hospital Quality Star Ratings With Random Measure Weights. *JAMA Netw Open*. Published July 03, 2025. doi:10.1001/jamanetworkopen.2025.19029

Data
Data available: Yes
Data types: Data (not involving human participants)
How to access data: Supplemental Tables 1,2,3 with study results have our data to share. The remaining study data is publicly available already on data.Medicare.gov.
When available: With publication

Supporting Documents
Document types: None

Additional Information
Who can access the data: Data available to anyone requesting data or downloading the supplements.
Types of analyses: Data can be used for any further analysis
Mechanisms of data availability: Data available to anyone requesting data or downloading the supplements.
